# Effect of Cetuximab-Conjugated Gold Nanoparticles on the Cytotoxicity and Phenotypic Evolution of Colorectal Cancer Cells

**DOI:** 10.3390/molecules26030567

**Published:** 2021-01-22

**Authors:** Ralph El Hallal, Nana Lyu, Yuling Wang

**Affiliations:** Department of Molecular Sciences, Macquarie University, Sydney, NSW 2109, Australia; ralph.el-hallal@students.mq.edu.au (R.E.H.); nana.lyu@students.mq.edu.au (N.L.)

**Keywords:** epidermal growth factor receptor, colorectal cancer, cetuximab, cytotoxicity, gold nanoparticles, surface-enhanced Raman scattering/spectroscopy, phenotypes

## Abstract

Epidermal growth factor receptor (EGFR) is estimated to be overexpressed in 60~80% of colorectal cancer (CRC), which is associated with a poor prognosis. Anti-EGFR targeted monoclonal antibodies (cetuximab and panitumumab) have played an important role in the treatment of metastatic CRC. However, the therapeutic response of anti-EGFR monoclonal antibodies is limited due to multiple resistance mechanisms. With the discovery of new functions for gold nanoparticles (AuNPs), we hypothesize that cetuximab-conjugated AuNPs (cetuximab-AuNPs) will not only improve the cytotoxicity for cancer cells, but also introduce expression change of the related biomarkers on cancer cell surface. In this contribution, we investigated the size-dependent cytotoxicity of cetuximab-AuNPs to CRC cell line (HT-29), while also monitored the expression of cell surface biomarkers in response to treatment with cetuximab and cetuximab-AuNPs. AuNPs with the size of 60 nm showed the highest impact for cell cytotoxicity, which was tested by cell counting kit-8 (CCK-8) assay. Three cell surface biomarkers including epithelial cell adhesion molecule (EpCAM), melanoma cell adhesion molecule (MCAM), and human epidermal growth factor receptor-3 (HER-3) were found to be expressed at higher heterogeneity when cetuximab was conjugated to AuNPs. Both surface-enhanced Raman scattering/spectroscopy (SERS) and flow cytometry demonstrated the correlation of cell surface biomarkers in response to the drug treatment. We thus believe this study provides powerful potential for drug-conjugated AuNPs to enhance cancer prognosis and therapy.

## 1. Introduction

Colorectal cancer (CRC) has been the third leading cause of cancer-related mortality and the fourth commonly diagnosed cancer worldwide. Nearly two million new cases and about one million deaths occurred in 2018 [1]. The metastatic disease accounts for up to 20% of newly diagnosed patients and further develops in 50% of CRC cases [2,3]. The clinical outcome of patients with metastatic CRC (mCRC) has been improved by the introduction of cetuximab and panitumumab, two monoclonal antibodies targeting the epidermal growth factor receptor (EGFR) [3,4]. EGFR, the target of these drugs, plays a key role in the development and progression of CRC by promoting a variety of functions including proliferation, survival, invasion, or immune evasion [5]. Cetuximab binds to the extracellular domain of EGFR and prevents ligand-induced activation of intracellular pathways, such as Raf/MEK/ERK and PI3K/Akt cascades, leading to growth suppression and cell death [6]. In addition to their effect on ligand binding, cetuximab can promote EGFR internalization and subsequent degradation, thus decreasing the cell surface level of EGFR [5,6]. However, the overexpression of EGFR in approximately 80% of CRC failed to predict a therapeutic response to anti-EGFR treatment when used in clinic [6,7]. Only about ten percent of genetically unselected patients experience tumor regression when treated with anti-EGFR antibodies. The KRAS-mutant gene has been demonstrated to be intrinsically resistant to EGFR-targeting antibodies, which is called primary resistance. While some reports suggested that BRAF mutation status has clear prognostic value in mCRC, the predictive value of BRAF mutation status remains controversial for response to cetuximab treatment [8,9,10]. Moreover, nearly all patients whose tumor initially respond to cetuximab treatment, eventually become refractory, which refers to acquired resistance [3,6,11]. It was reported that aberrant activation of alternative receptors, such as human epidermal growth factor receptor 2 (HER2) and human epidermal growth factor receptor 3 (HER3) overexpression, is one of the molecular mechanisms for the resistance to anti-EGFR treatment [12,13]. Typically, HER3, one member of EGFR family (EGFR, HER2, HER3, and HER4), plays a significant role in the formation of a heterodimer with EGFR on the surface of CRC cells, activating the intracellular signaling pathway when a ligand binds to the receptor [14,15]. The overexpression of HER3 in 30–80% of mCRC has been associated with the resistance to EGFR inhibitor [16,17,18].

Additionally, the overexpression of epithelial cell adhesion molecule (EpCAM), a 40-kDa glycoprotein expressed in 85% of colorectal carcinoma was reported to enhance the proliferative and invasive capacities of tumors and can inhibit differentiation and promote proliferation [19]. It was also suggested that EpCAM expression may be associated with CRC carcinogenesis, while the loss of EpCAM expression can be correlated with the progression, metastasis, and poor prognosis of CRC, which makes EpCAM a useful biomarker for the clinical diagnosis of CRC [14]. Another important surface biomarker, melanoma cell adhesion molecule (MCAM), also called CD146, is a known tumor suppressor. Previous studies reported that the reduced MCAM expression promoted tumorigenesis and cancer stemness in CRC [20,21]. Therefore, HER3, EpCAM, and MCAM biomarkers have shown great potential as prognostic markers in CRC for an effective analysis.

Gold nanoparticles (AuNPs) have found a wide range of biomedical applications (e.g., drug delivery, diagnostics, biosensing, bio-imaging, and theranostics) due to their appealing features, including high biocompatibility and facile conjugation to biomolecules. Previous results have shown that drugs conjugated with AuNPs may significantly increase chemosensitivity and delivery efficacy in a variety of cancer types including colorectal cancer, oral squamous cell carcinoma, pancreatic cancer, breast cancer, and prostate cancer [22,23,24,25,26,27]. In recent years, an increasing number of studies were performed on the interaction of cetuximab-conjugated AuNPs (cetuximab-AuNPs) against EGFR-overexpressing cancers. It was shown that cetuximab-AuNPs displayed enhanced EGFR endocytosis and the subsequent suppression of downstream signaling pathway, leading to the inhibition of cell proliferation and the acceleration of apoptosis compared to AuNPs or cetuximab alone [28,29,30,31]. These findings suggest that AuNPs have promising potential to be used as drug carriers to increase the therapeutic efficiency of monoclonal antibodies. Moreover, cetuximab-AuNPs could be added to the standard chemotherapy and radiotherapy, where the enhanced uptake by specific targeting, and the subsequent improved efficacy of the therapeutic agents/radiation could be a viable approach for the treatment of cancers with EGFR overexpression [32,33,34,35,36,37,38,39,40]. Nevertheless, the size effect of AuNPs on cytotoxicity and the phenotypic evaluation of EGFR-overexpressing cancers have not been well documented. Thus, investigating the cytotoxicity effect of AuNPs and cetuximab-AuNPs with different sizes as well the phenotypic evaluation of cancer biomarkers expression, will advance the applications of AuNPs in cancer prognosis and therapy.

Furthermore, the unique optical properties conferred by their localized surface plasmon resonance (LSPR) [31,41,42,43,44] make AuNPs attractive for surface-enhanced Raman scattering/spectroscopy (SERS), which is a surface sensitive technique that measures Raman scattering of molecules adsorbed on the surface of the metallic (plasmonic) nanoparticles [45]. SERS has the ability to measure and detect single molecule while having multiplexing capabilities [46]. These features make SERS a powerful technique for sensitively and simultaneously detecting multiple biomarkers [41,47,48]. In this work, AuNPs and cetuximab-AuNPs with different sizes were synthesized and it was desired to study their cytotoxicity on BRAF-mutant HT-29 CRC cells (RAS wild-type) to advance the understanding of treatment response for cancers with BRAF mutation. In addition to cytotoxicity study, the phenotypic evolution of the cell surface markers, including EpCAM, MCAM, and HER3, were also studied for their responses to cetuximab and cetuximab-AuNPs treatment, by taking advantage of AuNPs for therapeutics and enhancement in Raman scattering (SERS nanotags). Analyzing the phenotypic evolution during treatment will provide insights on the resistance mechanism of drug treatment.

## 2. Results and Discussion

### 2.1. Synthesis and Characterization of Gold Nanoparticles

Although it has been reported that the cytotoxicity and cellular uptake of AuNPs are size-dependent to a large extent [49,50,51], AuNPs at the size of 25, 40, 60, and 80 nm were selected in this study by considering both the cellular uptake efficiency and SERS activity.

**Figure 1:**
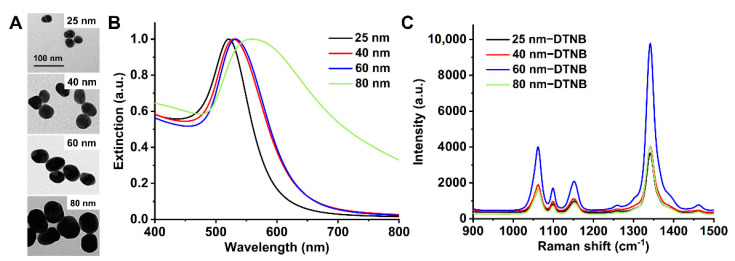
Characterization of gold nanoparticles (AuNPs). (**A**) Transmission electron microscopy (TEM) images of AuNPs at sizes of 25, 40, 60, and 80 nm; (**B**) Normalized UV-Vis spectra of AuNPs at different sizes; (**C**) surface-enhanced Raman scattering/spectroscopy (SERS) spectra of Raman reporter 5,5′-dithiobis-(2-nitrobenzoic acid) (DTNB) on AuNPs at different sizes (25, 40, 60, and 80 nm).

The size and morphology of the synthesized particles were examined by transmission electron microscopy (TEM). The TEM images of bare AuNPs displayed quasi-spherical particles with the sizes of 25, 40, 60, and 80 nm (Figure 1A). The surface plasmon resonance (SPR) of the AuNPs with different sizes were characterized with UV-Vis absorption spectroscopy (Figure 1B), in which the maximum absorption peak shifts from 521 to 560 nm with an increase in particle size from 25 to 80 nm. The largest AuNPs (80 nm) gave a much broader peak, indicating the wide size distribution of AuNPs (80 nm) compared to AuNPs at smaller sizes. SERS spectra of Raman reporter 5,5′-dithiobis-(2-nitrobenzoic acid) (DTNB) on AuNPs at different sizes (25, 40, 60, and 80 nm) were tested as shown in Figure 1C. Also, 60 nm AuNPs displayed the highest Raman enhancement for DTNB due to the localized surface plasmon resonance (LSPR) [52,53,54].

Meanwhile, it was observed that 80-nm AuNPs were more prone to aggregate during the preparation of Raman molecule-coated AuNPs and cetuximab-AuNPs (Figure S1, Supplementary Material). Since AuNPs synthesized in this study were based on citrate reduction and stabilization [55], the citrate-capped AuNPs are known to be electrostatically stabilized due to adsorption of citrate trianions (citrate^3−^) where the steric repulsion of citrate layers plays important role in stabilizing AuNPs [56]. During the preparation of Raman molecules coated AuNPs, if Raman molecules cannot be quickly anchored on the surface of AuNPs to form the self-assembled monolayer (SAM), the AuNPs can approach each other in close proximity, where van der Waals force dominates to induce aggregation. Since van der Waals force scales linearly with the size of AuNPs [57], this may explain that larger AuNPs (80 nm) are more susceptible and ready to aggregate during the process of functionalization with repeated centrifugations and particle dispersion steps [56,58,59,60].

Thus, AuNPs at the size of 25, 40, and 60 nm were used in cytotoxicity study. Because of the higher SERS activity of AuNPs at the size of 60 nm, it was used for profiling of the cell surface biomarkers after coating with Raman reporter molecules and conjugating with antibodies.

The size distribution of AuNPs (25, 40, and 60 nm) was also examined with dynamic light scattering (DLS), where the average hydrodynamic size was 32.2, 59.7, and 70.8 nm, respectively (Figure S2 and Table S1), which is slightly higher than that measured with TEM due to the hydration layer surrounding a particle in the hydrodynamic diameter [61,62]. The size distribution of AuNPs before and after conjugating with cetuximab was also measured with DLS for 60-nm AuNPs (Figure S2B), which shows the increased size from 70.8 to 88.2 nm, indicating the conjugation of cetuximab on the AuNPs.

The zeta potential of AuNPs and cetuximab-AuNPs was measured by electrophoretic light scattering (ELS), in which the citrate-capped AuNPs are negatively charged (Table S1). It was reported that zeta potential of AuNPs is size-dependent [63,64]; however, the increase of zeta potential is not linear to the size increment, the difference of zeta potential between AuNPs with sizes of 25 (−25.6 mV) and 40 nm (−33.2 mV) is a bit more significant than that for 40 and 60 nm (−33.1 mV). The zeta potential of AuNPs increased slightly (became less negative) from −33.1 to −27.7 mV upon binding with cetuximab, which has an isoelectric point of 8.5 [31] and was positively charged in the neutral phosphate buffered saline (PBS) buffer (pH 7.4).

### 2.2. Effects of Cetuximab-AuNPs on the Cell Viability

Selection of a suitable cytotoxicity assay is vital to accurately evaluate cell viability without the toxic effects on cells from the dye [65]. Cell counting kit-8 (CCK-8) assay was used as a colorimetric method for testing cell viability to evaluate the cytotoxic effect of cetuximab and cetuximab-AuNPs on HT-29 cells [66,67,68]. Since the cytotoxicity of AuNPs and cetuximab-AuNPs on cells is dose-dependent [31,69], the cell viability was investigated by applying two different concentrations of cetuximab. The conjugation amount of cetuximab in cetuximab-AuNPs ranged from 12.14 to 12.43 µg/mL measured by BCA protein assay for the AuNPs with sizes from 25 to 60 nm, where the amount of cetuximab in the final cetuximab-AuNPs complex was consistent for different sizes of AuNPs. Taking AuNPs (60 nm) as an example, the conjugation number of cetuximab molecules on each AuNP was estimated to be 405 (N_Cetuximab_:N_AuNP_ ≈ 405). The detailed calculation for the number of cetuximab molecules on AuNPs was provided in the Supplementary Material.

Since cetuximab-AuNPs (coated with 0.05% *w*/*v* BSA) were used to treat the cells in this study, the stability of the conjugates in the cell culture media (McCoy’s 5A media supplemented with 10% (*v*/*v*) FBS) was further investigated over 72 h by monitoring UV-Vis spectrum of cetuximab-AuNPs (Figure S3A). The average decrease of UV-Vis absorbance over 72 h is around 0.03 (a.u.) per 24 h from the initial absorbance of 1.38 (a.u.). Meanwhile, neither peak shifts nor shoulder peaks are observed in the absorption spectra, illustrating that cetuximab-AuNPs are stable in the cell culture media over 72 h. The stability was also analyzed with DLS (Figure S3B), where the particle size distribution exhibits no significant change over 72 h. The stability of antibody-conjugated AuNPs in the biological system has been illuminated in our previous study [70], which illustrated that increasing the protein thickness by antibody functionalization, protein coating, or inclusion of extra protein components in the detection environments can improve the stability of the particles.

**Figure 2:**
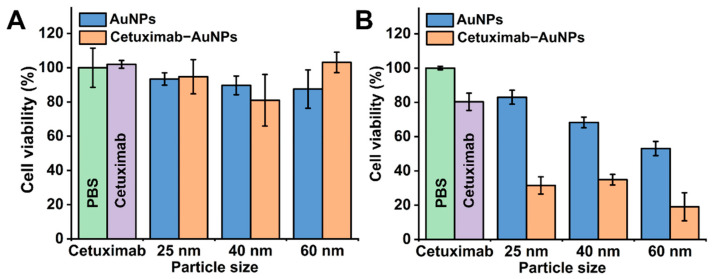
Cytotoxicity study by CCK-8 assay for HT-29 cells treated with cetuximab, AuNPs and cetuximab-AuNPs with sizes of 25, 40, and 60 nm for 48 h. (**A**) Low concentration of cetuximab (5 µg/mL); (**B**) High concentration of cetuximab (10 µg/mL). The cells treated with phosphate buffered saline (PBS) only and with cetuximab only were shown as green bar and purple bar, respectively. Error bar represents standard deviation (SD) of 5 replicates in 96-well plate.

For the cytotoxicity study, the required amount of cetuximab-AuNPs were calculated and concentrated to 20 µL to ensure that the same amount of cetuximab was added in each well of the 96-well plate. As indicated in Figure 2A with the cetuximab concentration of 5 µg/mL, the cells without cetuximab treatment (treated with PBS) were set as control with 100% viability, no significant decrease in cell viability was observed in the cetuximab, AuNPs, or cetuximab-AuNPs treated groups for 48 h. The comparison between AuNPs (25, 40, or 60 nm) alone and the corresponding cetuximab-AuNPs counterparts showed no significant difference. This may be attributed to the insufficient concentration of cetuximab (5 µg/mL) for inducing a response from HT-29 cancer cells. Therefore, an increased concentration of both cetuximab (10 µg/mL) and AuNPs was applied to evaluate the effects of AuNPs with different sizes for treating HT-29 cells (Figure 2B).

When the amount of cetuximab and cetuximab-AuNPs was doubled, cetuximab (10 µg/mL) had a significant effect on cell cytotoxicity by decreasing the cell viability by 19.7%, in which the cell viability was 80.3% ± 5.0% compared to the PBS control (100.0% ± 1.0%), as indicated in Figure 2B. Meanwhile, the doubled amount of AuNPs also showed significant cytotoxicity to HT-29 cells, where the cell viability was 83.0% ± 4.0%, 68.3% ± 3.1%, and 53.0% ± 4.1% after treating with AuNPs at sizes of 25, 40, and 60 nm, respectively. Due to the cytotoxicity of both cetuximab and AuNPs, cetuximab-AuNPs showed increased synergistic cytotoxicity compared to the individual cetuximab and AuNPs.

The effects of cetuximab-AuNPs decreased the cell viability significantly by greater than 60% for each size of AuNPs (25, 40, and 60 nm), where AuNPs at a size of 60 nm had the greatest effect on cell viability of HT-29 cells, which was decreased to 19.1%. The highest impact of 60 nm AuNPs on cell cytotoxicity can be attributed to the higher cell uptake efficiency of 60 nm AuNPs and the subsequent internalization and degradation of EGFRs in lysosome. Previous studies illustrated that AuNPs with a size of 40–60 nm have the highest cellular uptake efficiency, which endows AuNPs not only as simple carriers for biomedical applications but also play an active role in mediating biological effects [49,50,51,71]. Furthermore, it was demonstrated that the internalization and subsequent degradation of EGFRs in lysosome are important determinants for inhibiting cell proliferation and inducing apoptosis [3,5,72,73]. Taken together, these two effects have resulted in the highest cytotoxicity of cetuximab-AuNPs (60 nm) for treating HT-29 cells. The apoptosis study with annexin-FITC/PI analysis (Figure S4 and Table S2) further demonstrated that cetuximab-AuNPs significantly accelerated HT-29 cells apoptosis (27.86% apoptotic cells), compared with cetuximab (15.68%) and AuNPs (12.22%), which was probably due to the enhanced EGFR endocytosis and the subsequent suppression of downstream signaling pathway [31]. Conjugation of cetuximab to AuNPs enhances cetuximab induced endocytosis of EGFR which may have altered the cellular processes associated with ligand binding [74]. It was reported that the cellular uptake of AuNPs was significantly affected by the nanoparticle size, concentration, and the cell type [51,71,75]. The cytotoxicity study in this contribution showed that the cytotoxicity of AuNPs is dose and size-dependent for HT-29 cells, and cetuximab conjugated to AuNPs demonstrated significantly improved cytotoxicity compared to cetuximab used alone.

### 2.3. Profiling the Cell Surface Markers in Response to Cetuximab and Cetuximab-AuNPs Treatment

**Figure 3:**
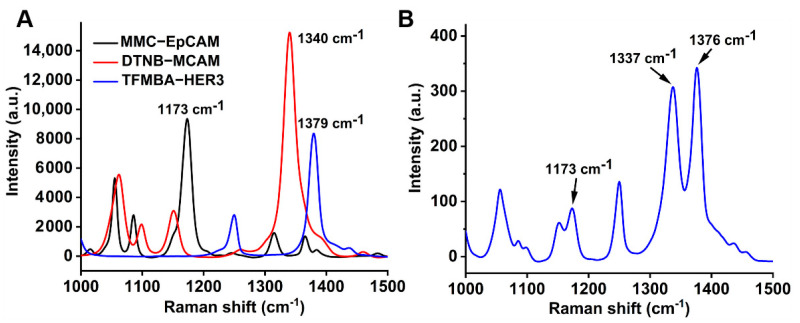
(**A**) SERS spectra of individual SERS nanotags; (**B**) SERS spectra of 3-plex SERS nanotags. The arrows indicate the characteristic peak of each nanotag for detecting the corresponding biomarker.

Although the use of cetuximab has greatly improved the clinical outcomes of metastatic CRC, the discovery of resistance to anti-EGFR monoclonal antibodies stimulated interest in the study of drug resistance, including abnormal molecules in the EGFR pathway, abnormal activations between the paralleled pathways, and other mechanisms [12]. In this study, the surface markers EpCAM, MCAM, and HER3 of HT-29 cells after treating with cetuximab or cetuximab-AuNPs, were profiled with multiple SERS nanotags, which were coated with the individual Raman reporter (MMC, DTNB, or TFMBA) and conjugated with the corresponding antibody, to provide a unique Raman fingerprint for each surface marker. As indicated in Figure 3A, MMC at 1173 cm^−1^ is for EpCAM, DTNB at 1340 cm^−1^ is for MCAM, and TFMBA at 1379 cm^−1^ is for HER3. The characteristic peaks for Raman reporters in the multiplex detection (Figure 3B) were used to profile the plot of frequency vs. signal distribution [18].

**Figure 4:**
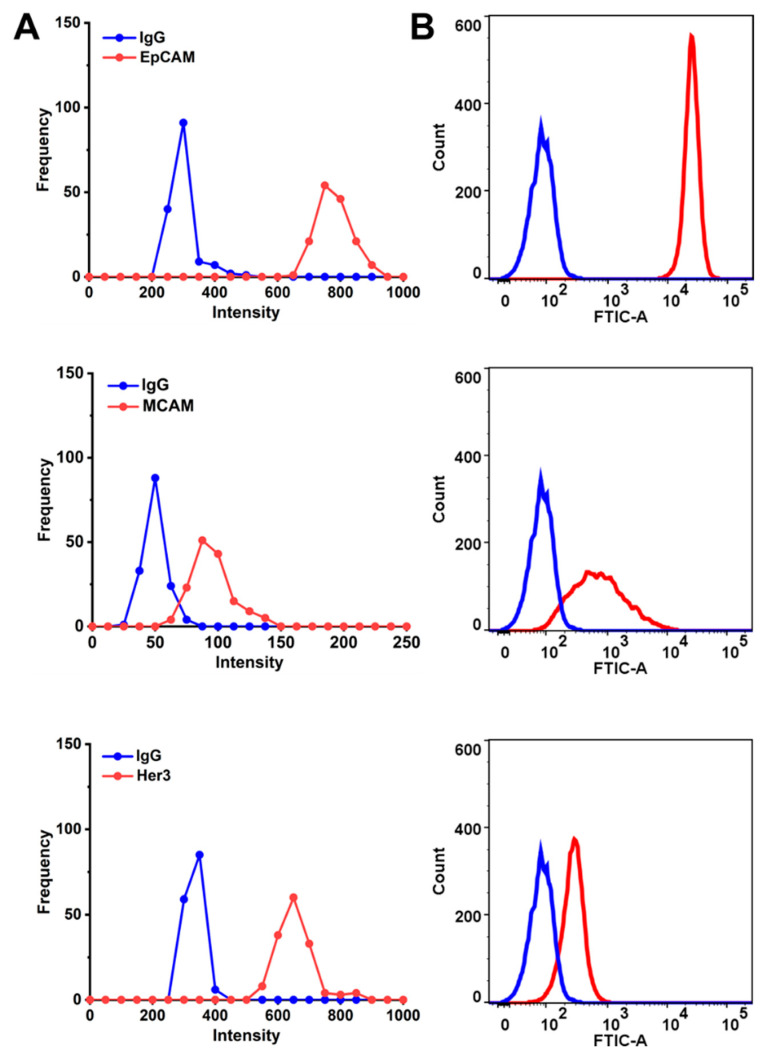
Phenotyping of the cell surface biomarkers. (**A**) Raman signal frequency distribution and (**B**) flow cytometry for cell surface markers (EpCAM, MCAM, and HER3) before drug treatment.

The cell surface biomarker’s expression (signal) distribution curve (Figure 4A) was obtained by extracting the intensity of the characteristic peaks for the respective marker from the 150 Raman measurements for each sample, then the frequency for each intensity range was counted, this allows the profiling of the expression levels for multiple markers [18]. It was hypothesized that the wider signal distribution of the respective surface marker represents the more heterogeneous phenotypes. The peak shift of the frequency-intensity plot indicated the change in the expression level for the respective marker. Isotype-matched immunoglobulins (IgG) antibodies were used as the control. Flow cytometry was used as a standard method to validate the SERS signal intensity distribution for the respective surface marker (Figure 4B). As illustrated in Figure 4, EpCAM and MCAM have shown higher expression level on the surface of HT-29 cells, while HER3 showed relatively low expression on the cells, the percentage of cells expressing the respective biomarkers is also demonstrated in Figure S5.

**Figure 5:**
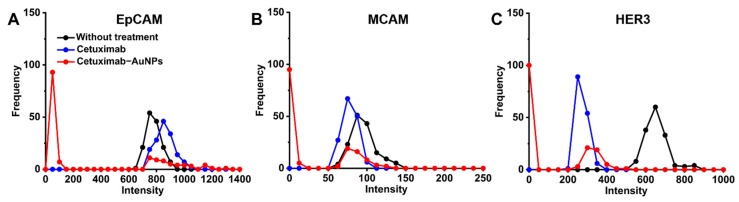
The evolution of surface marker expression after treating with cetuximab and cetuximab-AuNPs. Raman signal intensity distribution for cell surface markers EpCAM (**A**), MCAM (**B**), and HER3 (**C**).

The cell signature is defined as the relative expression levels of three biomarkers. In our study, we studied three cell surface markers simultaneously to profile the cell signature and investigate its evolution during drug treatment. Moreover, the intensity distribution shifts to the lower signal for cell surface biomarkers MCAM and HER3 on HT-29 cells (Figure 5), which demonstrates the decreased expression level in response to cetuximab treatment compared to the untreated cells, while EpCAM showed no significant change in the expression level in response to cetuximab or cetuximab-AuNPs treatment detected by flow cytometry (Figure S5). However, SERS detection demonstrated a slight shift to the lower signal and the higher heterogeneity of the EpCAM expression (Figure 5).

The application of cetuximab-AuNPs would increase the endocytic capacity and hence the degradation of EGFR. The absence of EGFR plays a key role in the decreased activation of cell signaling pathway after drug treatment, thus leading to the inhibition of surface markers expression. For the cells treated with cetuximab-AuNPs, the expression of MCAM and HER3 were decreased, while the EpCAM expression showed no significant decrease but higher heterogeneity which may be a good sign for further exploration on the genetic profiles to determine the signaling pathway for explaining the cells phenotype. As the phenotype was only studied at a single time point for drug treatment (*t* = 48 h). It is suggested that a systematically dynamic study on the phenotype will help to enhance our understanding for the potential signaling pathway and the related drug resistance mechanism. This further substantiates the evidence that AuNPs have effects on cytotoxicity and the related surface biomarkers expression on cancer cells and may be a promising therapeutic solution to enhance prognostic analysis for colorectal cancer.

## 3. Conclusions

In summary, different sizes of AuNPs were proven to induce cytotoxicity for colorectal cancer HT-29 cells when conjugated with cetuximab. Among them, AuNPs of 60 nm conjugated with cetuximab showed the highest cytotoxicity for HT-29 cells when compared to the free cetuximab in the CCK-8 assay. To further investigate the effects of cetuximab and cetuximab-AuNPs (60 nm) on the phenotypes of cells, the signature of cell surface markers EpCAM, MCAM, and HER3 were profiled with the multiple antibody conjugated SERS nanotags. The results showed the evolution of cell surface signature, in which not only the expression levels of MCAM and HER3 decreased significantly but also the cell surface signatures (the relative ratio of these markers) changed after being treated with cetuximab or cetuximab-AuNPs. Cetuximab-AuNPs had similar yet increased effects on the evolution trend of cell surface signatures, which may be attributed to the upregulation of the parallel pathways when the EGFR signaling pathway was inhibited by cetuximab, and the decreased expression of MCAM and HER3 may contribute to the complex mechanisms of resistance to anti-EGFR treatment. Therefore, this study provides a further promising outlook for treating cells by conjugating cetuximab on AuNPs and profiling the evolution of cell surface signatures by multiplex detection with SERS nanotags.

## 4. Materials and Methods

### 4.1. Chemicals

The Raman molecules 5,5′-dithiobis-(2-nitrobenzoic acid) (DTNB) and 7-mercapto-4-methylcoumarin (MMC) were obtained from Sigma-Aldrich, 2,3,5,6-tetrafluoro-4-mercaptobenzoic acid (TFMBA) was purchased from Tokyo Chemical Industry (TCI, Tokyo, Japan). Gold(III) chloride trihydrate (HAuCl_4_·3H_2_O) and sodium citrate tribasic dihydrate used for synthesis of gold nanoparticles (AuNPs) were purchased from Sigma-Aldrich. Monoclonal antibodies anti-EGFR (research grade cetuximab biosimilar, MAB9577), anti-EpCAM (MAB9601), anti-MCAM (MAB932), anti-HER3 (MAB3481), and IgG isotype control (MAB002) were purchased from R&D systems. The 3,3′-dithiobis(sulfosuccinimidyl propionate) (DTSSP) supplied by Sigma-Aldrich were used for conjugating antibodies to AuNPs. Micro BCA Protein Assay Kit (Thermo Scientific, Rockford, IL, USA) was used for analyzing the amount of protein conjugated to AuNPs. Cell Counting Kit-8 (CCK-8) purchased from Sigma-Aldrich were used for determining the cell viability in the cytotoxicity assay. Annexin V-FITC apoptosis detection kit (Cat No. APOAF, Sigma-Aldrich, St. Louis, MO, USA) was used to test the apoptosis of cells upon treatment. Cell line HT-29 were purchased from American Type Culture Collection (ATCC, Manassas, VA, USA).

### 4.2. Instruments

Transmission electron microscope (TEM, Philips CM10) was used to visualize the size and morphology of AuNPs. Zetasizer (Malvern, UK) was applied to examine the hydrodynamic size and zeta potential of AuNPs. UV-Vis spectroscopy (Cary 5000, Agilent, Santa Clara, CA, USA) was employed to check the optical characteristics of AuNPs. A Raman microscope equipped with a 785-nm wavelength laser (IM-52, Snowy Range Instruments) was utilized for SERS measurement. BD LSRFortessa X-20 flow cytometer (BD Biosciences, San Jose, CA, USA) was used to test the flow cytometry.

### 4.3. Synthesis of Gold Nanoparticles

Gold nanoparticles (AuNPs) were synthesized by citrate reduction of gold chloride with sodium citrate in aqueous solution as reported [55]. Briefly, 50 mL of HAuCl_4_ (0.01% *w*/*v*) was heated until boiling, then 0.75, 0.5, 0.35, and 0.25 mL of sodium citrate (1% *w*/*v*) was added, and the mixture was continuously boiled and stirred for 20 min to obtain AuNPs with diameter of 25, 40, 60, and 80 nm, respectively.

### 4.4. Preparation of SERS Nanotags

The antibody conjugated SERS nanotags (Ab-SERS nanotags) were prepared by functionalizing AuNPs with Raman reporter molecules and antibodies as previously reported [76]. Briefly, 10 µL of 1-mM Raman reporter molecules (DTNB, MMC, TFMBA) were added into 1 mL AuNPs (60 nm) suspension concentrated from 1.5 mL as-prepared AuNPs colloidal, the mixture was then incubated at room temperature (RT) for 5 h to form a complete self-assembled monolayer (SAM). Subsequently, the mixture was centrifuged at 5500 rpm for 8 min to remove the residual reactants, then re-dispersed in 300 µL of 0.1 mM PBS and incubated with DTSSP-linked antibody solution, which was prepared by adding 5 µL of 1 mg/mL DTSSP solution (dissolved in 5 mM sodium citrate buffer, pH 5.3) and 10 µL of 0.5 mg/mL antibody solution (anti-EpCAM, anti-MCAM or anti-HER3, dissolved in PBS). The mixture was incubated at RT for 30 min under shaking (300 rpm) and kept at 4 °C overnight. After overnight incubation, the products were centrifuged at 4 °C, 1000× *g* for 10 min to remove free antibodies. Then the SERS nanotags were re-dispersed in 300 µL of BSA (0.05% *w*/*v* in 0.1 mM PBS) and incubated at RT for 30 min under shaking (350 rpm) to block the non-specific binding sites.

The cetuximab-AuNPs used for treating cells were prepared with the above-mentioned protocol but without Raman molecules coating. The amount of conjugated cetuximab was determined by bicinchoninic acid (BCA) assay before BSA coating. The BSA assay was performed according to the manufacturer’s instruction.

### 4.5. Cell Viability Study by CCK-8 Assay

The amount of cetuximab in cetuximab-AuNPs (25, 40, and 60 nm) was determined by BCA assay. The cetuximab-AuNPs was centrifugated at 4 °C, 1200× *g* for 10 min, and re-dispersed in 20 µL of PBS (containing 1% *v*/*v* of FBS) to ensure the final amount of cetuximab added into each well (100 µL) of HT-29 cells was 0.5 µg (or doubled amount 1.0 µg) for the cell viability assay. The corresponding amount of bare AuNPs concentrated in 20 µL of PBS (containing 1% *v*/*v* of FBS) were used as control.

HT-29 cells were cultured in McCoy’s 5A medium (ATCC 30-2007) supplemented with 10% (*v*/*v*) of fetal bovine serum (FBS, Life Technologies, Grand Island, NY, USA) and 1% (*v*/*v*) of Penicillin-Streptomycin solution (P4333, Sigma-Aldrich, St. Louis, MO, USA) under standard cell culture conditions (37°C, 5% CO_2_). HT-29 cells were collected and suspended in cell culture media for seeding into 96-well plate with a density of 2000 cells in 100 µL/well. After incubating overnight, the cetuximab (0.5 µg/well, or 1 µg/well), AuNPs (25, 40, and 60 nm) and cetuximab-AuNPs (25, 40, and 60 nm) were added to the wells and then incubated (37 °C, 5% CO_2_) for 48 h. The cells treated with 20 µL of PBS were used as control with 100% cell viability. Then, 10 µL of CCK-8 solution was added to each well and incubated (37 °C, 5% CO_2_) for 2 h. Subsequently, the absorbance at 450 nm was recorded using microplate reader (SPECTROstar Nano, BMG Labtech). The cell viability was calculated by dividing the absorbance of the experimental wells with the absorbance of the control well. The cell viability study was performed in 5 replicates (5 wells for each type of treatment).

### 4.6. Cell Apoptosis Study with Annexin-FITC/PI Analysis

HT-29 cells were seeded with a concentration of 2 × 10^5^ cells per 2 mL of McCoy’s 5A/10% FBS media to each well of a 6-well plate. Then, AuNPs, cetuximab (10 µg/mL) and cetuximab-AuNPs (containing 10 µg/mL of cetuximab) were added to incubate with cells for 48 h, where cells treated with PBS were used as control. Cells were then harvested from the 6-well plate following trypsinization, washed gently with PBS 2 times, and collected by centrifugation. Cell apoptosis was analyzed with annexin V-FITC apoptosis detection kit (Cat No. APOAF, Sigma-Aldrich) according to the manufacturer’s instruction. Briefly, after re-dispersing the collected cells for each well in 200 µL of 1× binding buffer, 5 µL of annexin V FITC conjugate and 5 µL of propidium iodide (PI) solution were added to stain the cells for exactly 10 min at room temperature and protected from light. After incubation, the cells were analyzed immediately with the BD LSRFortessa X-20 flow cytometer.

### 4.7. Profiling of Cell Surface Markers

To profile the cell surface markers EpCAM, MCAM, and HER3 after cetuximab and cetuximab-AuNPs treatment, HT-29 cells were seeded in a 6-well plate with a density of 2 × 10^5^ cells/well in 2 mL cell culture media, the cells were treated with cetuximab (10 µg/mL) or cetuximab-AuNPs (containing 10 µg/mL of cetuximab) for 48 h. Then the cells were harvested, washed with PBS and dispersed in 200 µL buffer (PBS supplemented with 1% *v*/*v* FBS) to obtain 5 × 10^5^ cells/mL cells suspension for profiling cell surface markers by SERS nanotags and flow cytometry.

For cell labelling with Ab-SERS nanotags, the protocol was described as reported [18], 200 µL of 10^5^ cells (5 × 10^5^ cells/mL) were incubated with the mixture of three Ab-SERS labels (30 µL each) at 37 °C for 30 min under shaking (300 rpm), followed by gentle centrifuge at 400× *g* for 1 min and washing with 200 µL buffer. The washing step was repeated for 4 times. The samples were then re-suspended in 60 µL buffer and placed into a cuvette for SERS measurements. SERS spectra were recorded with a portable IM-52 Raman Microscope (Snowy Range Instruments). The 785-nm laser wavelength was used for excitation of Raman scattering. SERS spectra were obtained at 1 s integration time with an incident laser power of 70 mW. 150 of SERS spectra were acquired for each sample.

Flow cytometry for characterizing the cell surface markers were performed on BD LSRFortessa X-20 flow cytometer. Cells (2 × 10^5^ cells) re-suspended in 100 µL of FACS buffer (PBS containing 3% *v*/*v* FBS, 0.5% *w*/*v* BSA and 1 mM EDTA) were incubated with 0.25 µg of anti-EpCAM, anti-MCAM, or anti-HER3 mouse monoclonal antibody, or isotype-matched control at RT for 30 min under shaking (300 rpm). Then the cells were washed with 200 µL of FACS buffer for 2 times. The cells were then dispersed in 200 µL of 1 µg/mL secondary antibody (Alexa Fluor 488 goat anti-mouse IgG antibody, A-11001, Life Technologies) and incubated at RT for 30 min under shaking (300 rpm). After washing the cells to remove excessive secondary antibody, the cells were re-dispersed in 200 µL of FACS buffer for flow cytometry.

## Data Availability

Not applicable.

## Supplementary Materials

The supporting information are available online, Figure S1: UV-Vis absorption spectra of AuNPs (80 nm), AuNPs (80 nm) with DTNB and AuNPs (80 nm) conjugated with cetuximab, Figure S2: Hydrodynamic size distribution of AuNPs at the size of 25, 40 and 60 nm (A) and AuNPs (60 nm) before and after conjugation with cetuximab (B) measured by dynamic light scattering (DLS), Figure S3: (A) UV-Vis spectra of cetuximab-AuNPs over 72 h; (B) Size distribution of cetuximab-AuNPs measured by DLS over 72 h, Figure S4: Apoptosis analysis of HT-29 cells treated with PBS (A), AuNPs at the size of 60 nm (B), cetuximab (C) and cetuximab-AuNPs (60 nm) (D) for 48 h, Figure S5: Phenotyping of the cell surface biomarkers (EpCAM, MCAM and HER3 by flow cytometry before treatment (A), and after treatment with cetuximab (B) and cetuximab-AuNPs (60 nm) (C), Table S1: Hydrodynamic size of AuNPs measured by DLS and zeta potential measured by electrophoretic light scattering (ELS), Table S2: The apoptosis rate for HT-29 cells treated with PBS, AuNPs (60 nm), cetuximab and cetuximab-AuNPs (60 nm) for 48 h.

## Sample Availability

The sample of compounds are not available from authors.
