# Supplementary material for: Effect of Cetuximab-Conjugated Gold Nanoparticles on the Cytotoxicity and Phenotypic Evolution of Colorectal Cancer Cells
Source: Molecules. 2021 Jan 22;26(3):567. doi: 10.3390/molecules26030567 (PMC7865832; doi:10.3390/molecules26030567)
Supplement: Supplementary file 1 [file molecules-26-00567-s001.pdf]

Supplementary Material

Effect of cetuximab-conjugated gold nanoparticles on the cytotoxicity and phenotypic evolution of colorectal cancer cells

Ralph El Hallal[*§], Nana Lyu[*§] and Yuling Wang[*,✉]

[*] Correspondence: Department of Molecular Sciences, Macquarie University, Sydney, NSW 2109, Australia

[✉] Corresponding author: yuling.wang@mq.edu.au

[§] These authors contributed equally

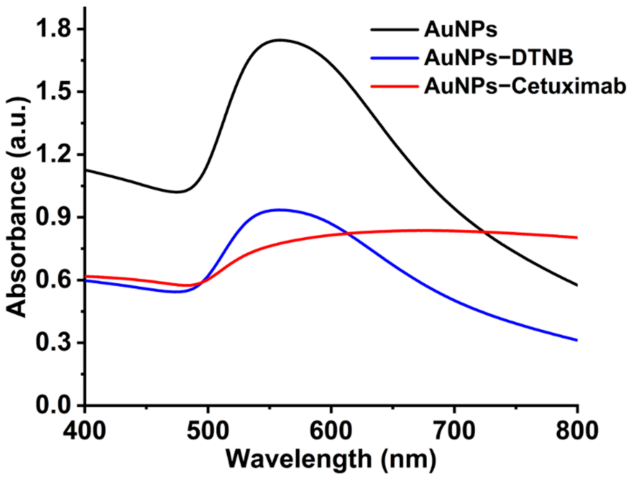

Figure S1. UV-Vis absorption spectra of AuNPs (80 nm), AuNPs (80 nm) with DTNB and AuNPs (80 nm) conjugated with cetuximab.

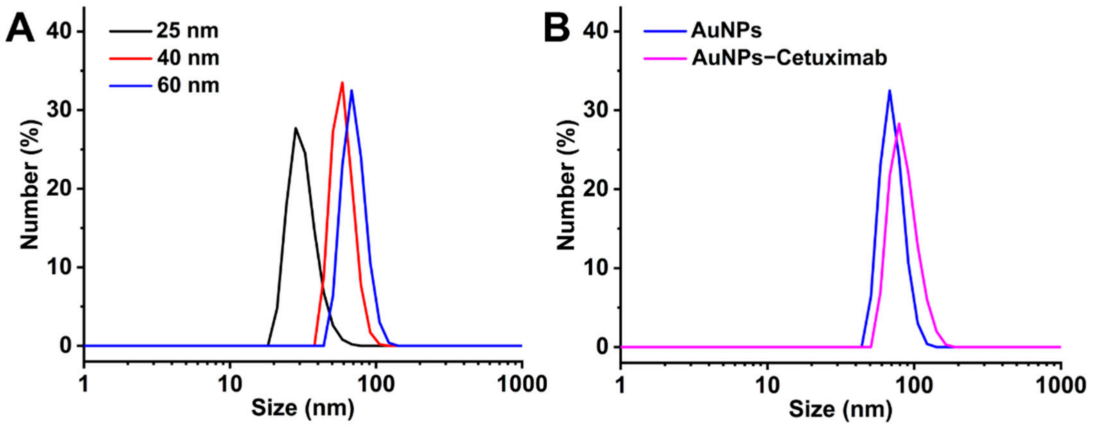

Figure S2. Hydrodynamic size distribution of AuNPs at the size of 25, 40 and 60 nm (A) and AuNPs (60 nm) before and after conjugation with cetuximab (B) measured by dynamic light scattering (DLS).

Table S1. Hydrodynamic size of AuNPs measured by DLS and zeta potential measured by electrophoretic light scattering (ELS) (n=3)

Sample	Size (nm)	Zeta potential (mV)
AuNPs (25 nm)	32.2 ± 0.5	-25.6 ± 0.5
AuNPs (40 nm)	59.7 ± 1.3	-33.2 ± 0.9
AuNPs (60 nm)	70.8 ± 1.4	-33.1 ± 0.3
AuNPs (60nm)-Cetuximab	88.2 ± 3.5	-27.7 ± 1.0

Calculation for the number of cetuximab conjugated on AuNPs

To calculate the number of cetuximab molecules on single AuNP, we first calculated the number of AuNPs in suspension. Taking AuNPs at the size of 60 nm as an example, we assume that 50 mL of 0.01% (w/v) $HAuCl_4 \cdot 3H_2O$ (Au^{3+}) was completely reduced to AuNPs (Au^0), the number of AuNPs from 1.5 mL as-prepared colloidal AuNPs is calculated as follows:

The number of moles for Au^{3+}:

$$n_{Au^{3+}} = \frac{Mass\ of\ HAuCl_4 \cdot 3H_2O}{Molecular\ weight\ (MW)\ of\ HAuCl_4 \cdot 3H_2O} = \frac{50\ mL \times 0.01 g/100mL}{393.8\ g/mol} = 1.27 \times 10^{-5}\ mol$$

Total mass of Au^0:

$$n_{Au^0} = n_{Au^{3+}}$$

$$m_{Au^0} = n_{Au^0} \times MW_{Au^0} = 1.27 \times 10^{-5} mol \times 196.97\ g/mol = 2.50\ \times 10^{-3} g$$

Volume of a single AuNP (60 nm, the radius is 30 nm):

$$V = \frac{4\pi r^3}{3} = \frac{4\pi}{3}(30\ nm)^3 = 1.13 \times 10^{-16}\ cm^3$$

Mass of a single AuNP:

$$m_{AuNP} = \rho_{Au} \times V_{AuNP} = 19.32\ g/cm^3 \times 1.13 \times 10^{-16}\ cm^3 = 2.18 \times 10^{-15}\ g$$

Total number of AuNPs in 50 mL:

$$N_{AuNPs} = \frac{m_{Au^0}}{m_{AuNP}} = \frac{2.50\ \times 10^{-3} g}{2.18 \times 10^{-15} g} = 1.15 \times 10^{12}\ AuNPs$$

Number of AuNPs in 1.5 mL:

$$N_{AuNPs\ in\ 1.5\ mL} = \frac{N_{AuNPs}}{50\ mL} \times 1.5\ mL = 3.45 \times 10^{10}\ AuNPs$$

To conjugate cetuximab on AuNPs, the concentration of AuNPs was concentrated from 1.5 mL to 0.3 mL. The number of AuNPs in 0.3 mL suspension is 3.45×10^{10} particles.

The concentration of cetuximab in cetuximab-AuNPs solution was determined with BCA assay, where 12.43 μg/mL of cetuximab was detected in 0.3 mL of AuNPs. The molecular weight of cetuximab is 145.5 kDa. The number of cetuximab on AuNPs is

calculated as follows:

Mass of cetuximab:

$$m_{cetuximab} = 12.43\ \mu g/mL \times 0.3\ mL = 3.73\ \mu g$$

The number of moles for cetuximab:

$$n_{cetuximab} = \frac{m_{cetuximab}}{MW_{cetuximab}} = \frac{3.37\ \times 10^{-6}\ g}{145.5 \times 10^{3}\ g/mol} = 2.32 \times 10^{-11}\ mole$$

Number of cetuximab:

$$N_{cetuximab} = n_{cetuximab} \times Avogadro\ constant$$
$$= 2.32 \times 10^{-11} mol \times 6.022 \times 10^{23}/mole$$
$$= 1.40 \times 10^{13}\ cetuximab$$

The number of cetuximab on each AuNP:

$$n_{cetuximab} = \frac{N_{cetuximab}}{N_{AuNPs}} = \frac{1.40 \times 10^{13}\ cetuximab}{3.45 \times 10^{10}\ AuNPs} \approx 405\ cetuximab/AuNP$$

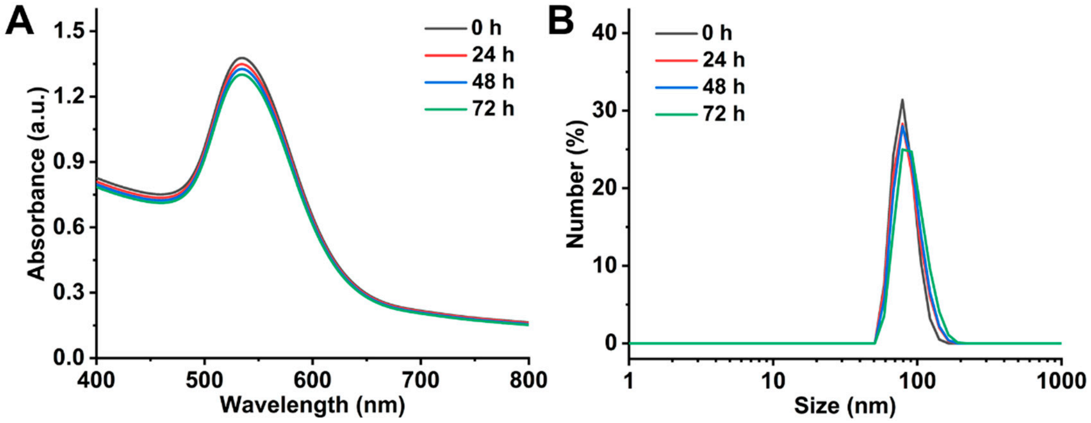

Figure S3. (A) UV-Vis spectra of cetuximab-AuNPs over 72 h; (B) Size distribution of cetuximab-AuNPs measured by DLS over 72 h.

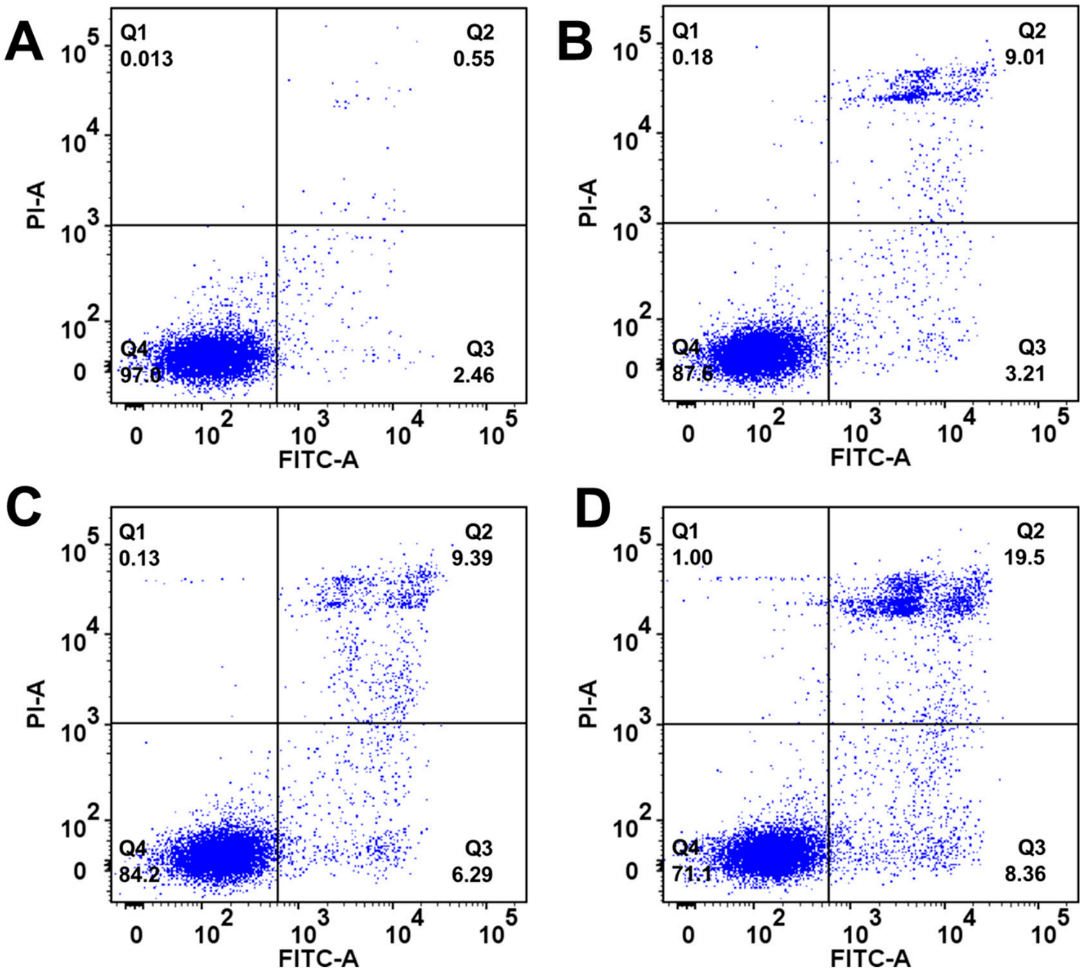

Figure S4. Apoptosis analysis of HT-29 cells treated with PBS (A), AuNPs at the size of 60 nm (B), cetuximab (C) and cetuximab-AuNPs (60 nm) (D) for 48 h. Quadrant gating: Q1: Annexin-/PI+ for necrotic cells; Q2: Annexin+/PI+ for late apoptotic cells; Q3: Annexin+/PI- for early apoptotic cells; Q1: Annexin-/PI- for viable cells.

Table S2. The apoptosis rate for HT-29 cells treated with PBS, AuNPs (60 nm), cetuximab and cetuximab-AuNPs (60 nm) for 48 h.

Sample	Early Apoptosis (%)	Late Apoptosis (%)	Apoptosis Rate (%)
Control	2.46	0.55	3.01
AuNPs	3.21	9.01	12.22
Cetuximab	6.29	9.39	15.68
Cetuximab-AuNPs	8.36	9.5	27.86

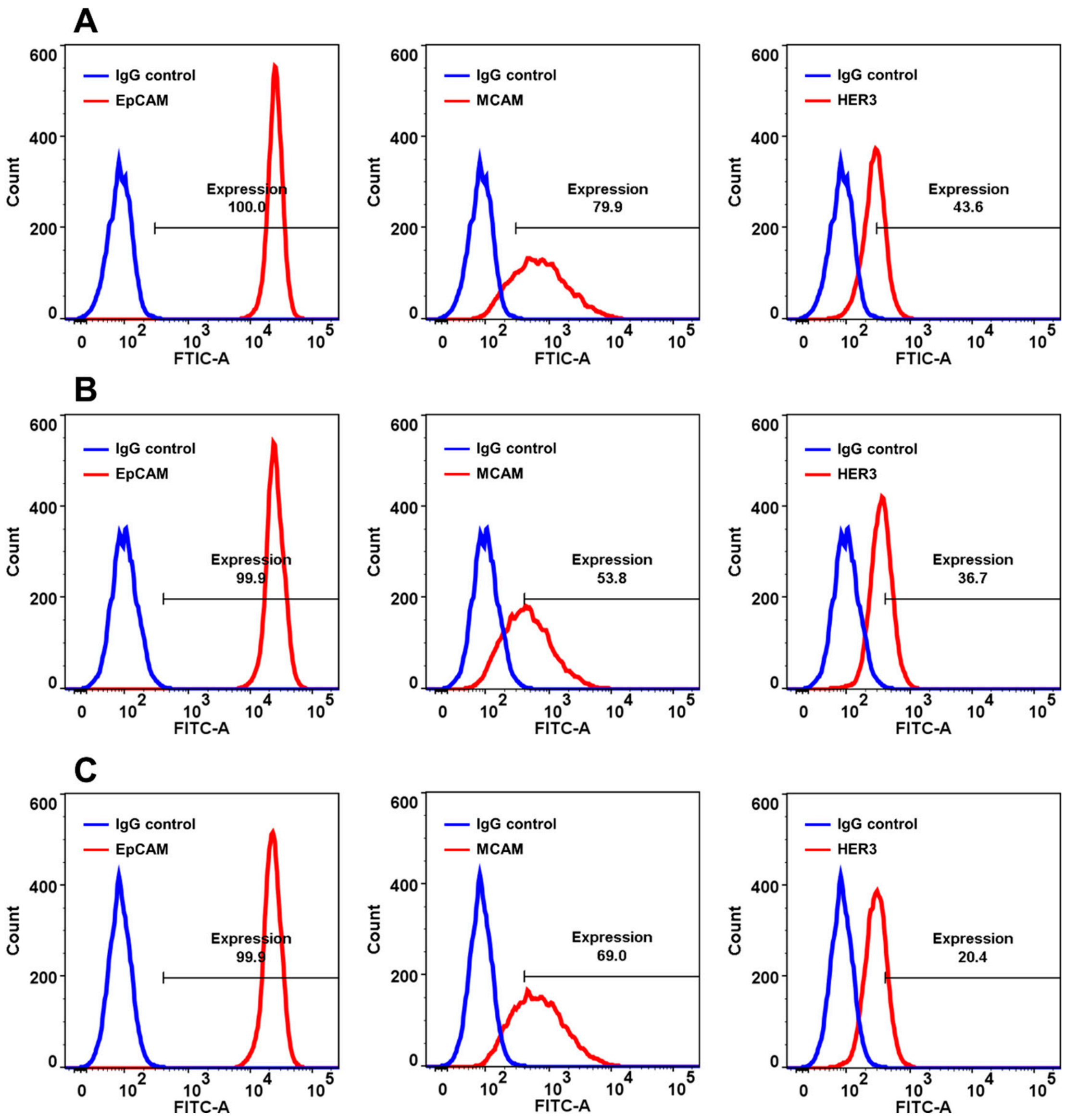

Figure S5. Phenotyping of the cell surface biomarkers (EpCAM, MCAM and HER3 by flow cytometry before treatment (A), and after treatment with cetuximab (B) and cetuximab-AuNPs (60 nm) (C).
